# Efficacy and safety of boric acid as a preventive treatment against *Saprolegnia* infection in Nile tilapia (*Oreochromis niloticus*)

**DOI:** 10.1038/s41598-019-54534-y

**Published:** 2019-11-29

**Authors:** Shimaa E. Ali, Amr A. A. Gamil, Ida Skaar, Øystein Evensen, Harrison Charo-Karisa

**Affiliations:** 1 Worldfish, Cairo, Egypt; 2 Department of Hydrobiology, National Research Centre, Dokki, Giza, Egypt; 3 Faculty of Veterinary Medicine, Norwegian University of Life Sciences, Oslo, Norway; 4 Norwegian Veterinary Institute, Oslo, Norway

**Keywords:** Drug screening, Fungi

## Abstract

Saprolegniosis is a worldwide fungal-like infection affecting freshwater fishes and their eggs. Reports show high mortalities and subsequent economic losses annually from *Saprolegnia* infections. Most therapeutants against *Saprolegnia spp*. infections are inefficient and some have negative impact on the environment. In this study, we have investigated the ability of boric acid (BA) to prevent *Saprolegnia* infection in Nile tilapia (*Oreochromis niloticus*). BA inhibited radial growth of *Saprolegnia* hyphae *in vitro*. Complete *in vitro* growth inhibition was found at a concentration of ≥0.6 g/L. Inhibitory effects were also observed *in vivo* when Nile tilapia were experimentally challenged with *Saprolegnia* spores and followed over 10 days post challenge and under continuous exposure to different BA concentrations. No signs of saprolegniosis were observed in fish treated with BA at concentrations of 0.4 g/L and above. Comet assay revealed that BA has low toxicity in tilapia continuously exposed to concentrations of 0.2–0.6 g/L for 96 h. Additionally, no significant histomorphological changes were observed in BA-treated fish compared to non-treated controls. Alanine Aminotransferase (ALT) and Aspartate Aminotransferase (AST) enzyme levels indicated reduction in systemic tissue damage associated with *Saprolegnia* infection. This study demonstrates the potential of BA as a prophylactic measure against *Saprolegnia* infection in tilapia, and we recommend additional studies on environmental impact.

## Introduction

Saprolegniosis is a serious threat to the aquaculture industry worldwide. The pathogen, *Saprolegnia*, belongs to oomycetes and are classified as fungal-like organisms^1^. The disease causes considerable economic losses in wild fish populations and in aquaculture^2,3^. Moreover, *Saprolegnia* sp. spores are difficult to prevent entering aquaculture facilities through the intake water. *Saprolegnia parasitica* in particular is associated with losses in different fish species including Nile tilapia (*Oreochromis niloticus)*. It has been shown that *S. parasitica* can cause >95% cumulative mortalities in Nile tilapia under experimental conditions and that differences in pathogenicity exist among *S. parasitica* strains^4^.

For many years, malachite green (MG) was the main treatment for *Saprolegnia* infections, highly efficacious and affordable. But due to its teratogenic, mutagenic and carcinogenic effects, the use of malachite green was banned worldwide^5,6^. Instead, therapeutants such as formalin, bronopol, sodium chloride and hydrogen peroxide are currently used to prevent or control *Saprolegnia* infections^7–9^, however, none of them are comparable to malachite green in efficiency. As a result, saprolegniosis has become an increasing challenge for the aquaculture industry^10–12^. Recently, we proposed boric acid (BA) as an efficient prophylactic treatment for *Saprolegnia* infection in salmonid eggs and yolk sac fry^13^. The high hatchability in treated salmon eggs and the high survival in yolk sac fry provided documentation that BA was safe for use. The present study was conducted to evaluate BA as a preventive treatment against *Saprolegnia* infection in Nile tilapia. In addition, the safety for use of BA in Nile tilapia fish was also investigated.

## Results

### Molecular identification of *Saprolegnia* strain

Following PCR and sequencing, a sequence of 690 bp of the ITS rRNA gene was obtained (Supplementary Fig. S1). Sequence alignment and phylogenetic analysis revealed the isolated strain as *S. parasitica*, giving %99 sequence similarity to public available sequences of *S. parasitica* (Supplementary Fig. S2).

### *In vitro* activity of BA against the *Saprolegnia* isolate

**Figure 1:**
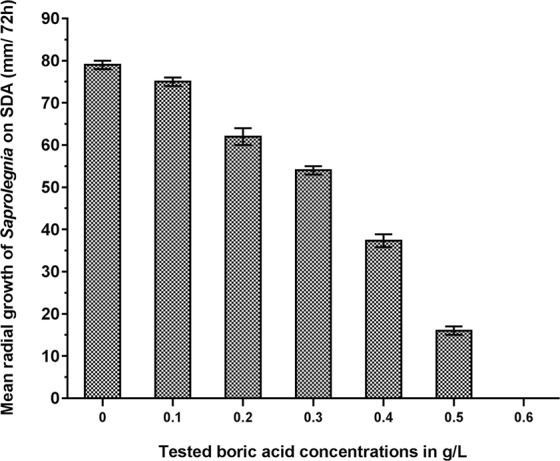
Inhibition of *Saprolegnia* growth on Sabouraud dextrose agar (SDA) following treatment with different BA concentrations. Partial inhibition occurred in all treatments ≤0.5 g/L while the complete inhibition was observed at concentrations 0.6 g/L and above.

A dose-dependent inhibition in the radial growth rates of *Saprolegnia* hyphae on Sabouraud Dextrose Agar (SDA) was observed. Treatment with 0.1 g/L concentration resulted in a partial but statistically significant (p = 0.006) inhibition of radial growth. 0.2 g/L gave a reduction of close to 40% (p < 0.0000) and increased gradually up to 0.6 g/L BA where no mycelial growth was observed (Fig. 1).

#### Toxicity testing and LC_50_

**Figure 2:**
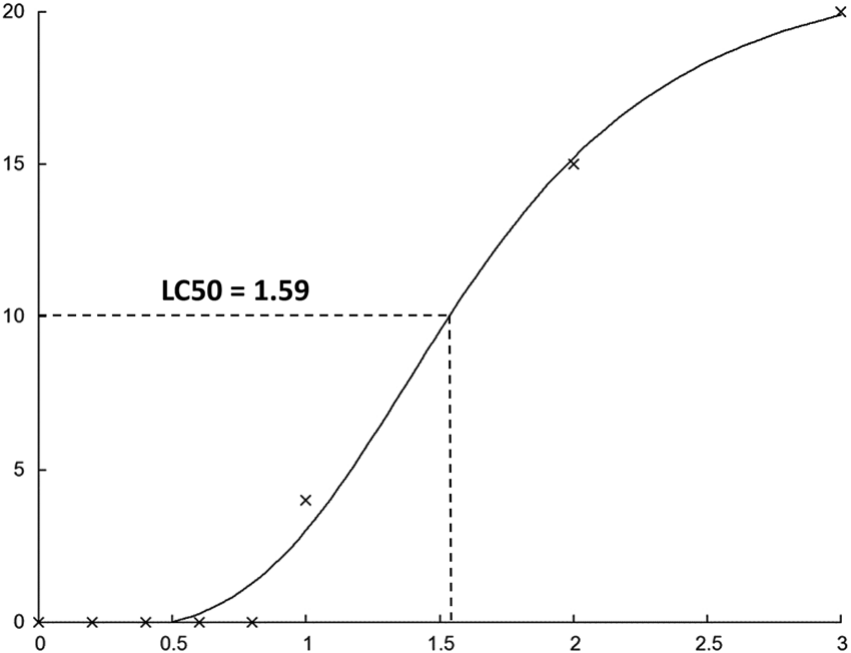
Dose response curve analysis and LC_50_ determination of BA treatment in Nile tilapia. LC_50_ was determined to be 1.59 g/L and calculated using an online software (http://www.ic50.tk).

Fish mortalities were recorded following 96 hours continuous exposure to BA at concentrations of 1, 2 and 3 g/L but not below 1 g/L (no water exchange over this period). Mortality rates differed with BA concentration, *i.e*. 20% at 1 g/L, 75% at 2 g/L, and 100% at 3 g/L. The MG treated positive control exhibited 100% mortalities by 84 hours post treatment (hpt). For both MG positive control and 3 g/L BA treatments, mortalities started at 60 hpt while for 1 and 2 g/L, mortalities started at 96 and 72 hpt, respectively (Table 1). A dose response curve was generated and the LC_50_ for Nile tilapia exposed to BA was determined to be 1.59 g/L (Fig. 2).

**Table 1:** Mortalities recorded in Nile tilapia following 96 hours continuous exposure to different boric acid (BA) concentrations and malachite green (MG).

Mortality	BA concentrations g/L								MG mg/L
	0	0.2	0.4	0.6	0.8	1	2	3	0.5
12 h	0/20	0/20	0/20	0/20	0/20	0/20	0/20	0/20	0/20
24 h	0/20	0/20	0/20	0/20	0/20	0/20	0/20	0/20	0/20
36 h	0/20	0/20	0/20	0/20	0/20	0/20	0/20	0/20	0/20
48 h	0/20	0/20	0/20	0/20	0/20	0/20	0/20	0/20	0/20
60 h	0/20	0/20	0/20	0/20	0/20	0/20	0/20	**10/20**	**6/20**
72 h	0/20	0/20	0/20	0/20	0/20	0/20	**5/20**	**5/10**	**10/14**
84 h	0/20	0/20	0/20	0/20	0/20	0/20	**10/15**	**5/5**	**4/4**
96 h	0/20	0/20	0/20	0/20	0/20	**4/20**	0/5	0	0
Total mortalities	0/20	0/20	0/20	0/20	0/20	4/20	15/20	20/20	20/20

Numbers of dead fish at different time points are presented in bold.

**Figure 3:**
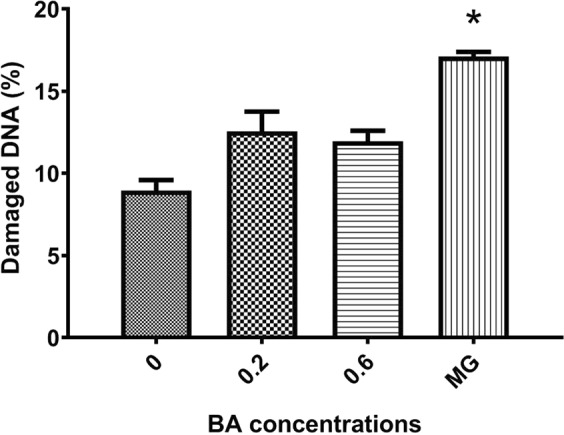
Percent of damaged DNA in blood after treatment with different boric acid (BA) concentrations. The percent of damaged nuclei was determined by counting 100 nuclei. Bars represent the average of 2 samples per concentration ± SD. Asterisk indicate statistical significance (*p < 0.05).

Since BA was previously shown to cause genotoxicity in fish, we analysed the genotoxic effect resulting from treatment with different BA concentrations with no reported mortalities using the comet assay. The results showed non-significant increase (about 2%) in the percentage of damage DNA in BA treatment compared to the control (Fig. 3). This was less than for the MG treated positive control, which showed 6% increase that was significantly (p < 0.05) different from the control group.

In contrast to the above results, histological evaluation (Supplementary Figs. S3–5) did not reveal differences between the non-treated control and BA (0.6 g/L) or MG treated fish. An exception was the gills of MG treated fish, which exhibited cellular hyperplasia in some parts (Supplementary Fig. S3c). This indicates that BA treatment at dosages of up to 0.6 g/L does not result in significant pathological changes.

### *In vivo* effect of BA treatment against *Saprolegnia* in Nile tilapia

**Figure 4:**
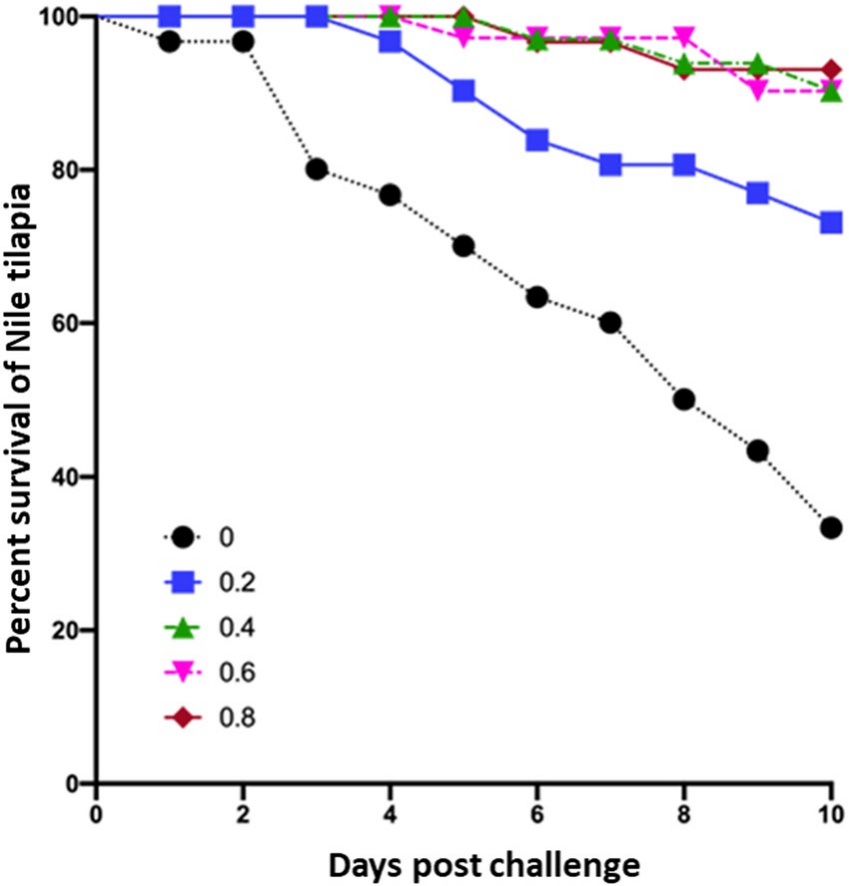
Effect of continuous exposure to boric acid (BA) treatment (from 0 to 0.8 g/L) on the survival of Nile tilapia experimentally infected with *S. parasitica*.

**Figure 5:**
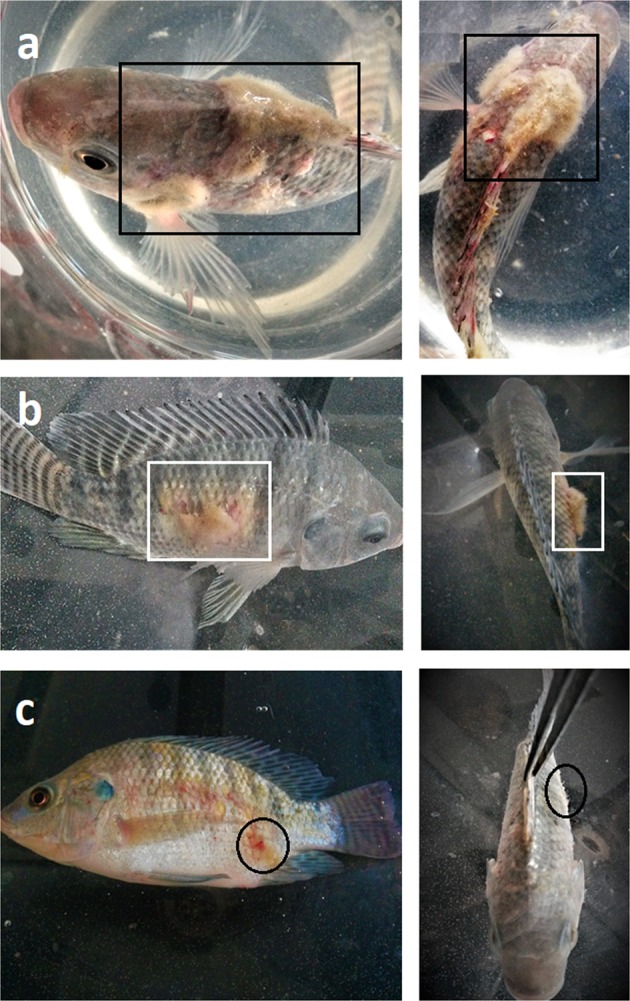
Tilapia experimentally infected with *S. parasitica* showing severe (black squares) and moderate (white squares) signs of infection in non-treated control group (**a,b**, respectively) while (**c**) is showing mild signs of infection (circles) associated with BA treatment at a concentration of 0.2 g/L. No signs of infection were observed in all BA treated groups at concentrations above 0.2 g/L.

Cumulative mortality, severity of lesions following *in vivo* infection with *S. parasitica* and effect of BA as preventive treatment against infestation over the 10-day period are shown in Table 2 and Fig. 4. PH was measured daily and the average value was 7.4 in the non-treated control group compared to 7.3 in the highest used BA concentration (0.8 g/L). Fish were examined for *Saprolegnia* associated lesions and the lesions were classified as either mild with scant mycelial growth on fish body (Fig. 5c), moderate, with obvious mycelial growth affecting single area or severe, with multiple obvious mycelial growth (Fig. 5b,a, respectively). The observed lesions included focal area of ulceration and necrosis in addition to appearance of cotton wool like growths on dorsal fin, operculum and abdomen (Fig. 5a). Untreated control fish started to die on day 3 and reached a maximum of 66.7% cumulative mortality by 10 days post infection (dpi). Among the treated fish, the highest survival (93.3%) was observed in fish treated with 0.8 g/L BA and lowest (73.3%) in fish treated with 0.2 g/L (Fig. 4, Table 3). Treatment with 0.2 g/L resulted in delayed onset of mortality and lower cumulative mortality, 26.7% by 10 dpi (Table 2). Fish treated with 0.4 and 0.6 g/L exhibited 10% mortality while those treated with 0.8 g/L had only 6.7% cumulative mortality by 10 dpi. None of the dead or surviving fish in these three groups showed signs of *Saprolegnia* infection. Log-rank test for equality of survivor function shows significant difference for 0–0.8 g/L (Chi-square = 46.03, p = 0.0000) and with Cox proportional hazard ratios shows a hazard ratio of 0.2–0.8 of 0.55 (p = 0.083), 0.11 (p < 0.000), 0.11 (p < 0.0000) and 0.07 (p < 0.0000), respectively. This documents reduced hazard ratios as the concentration of boric acid increases relative to control. Test for proportional hazard assumptions gives a Chi-square value of 0.98 (p = 0.3213) showing that the hazards are proportional for the model.

**Table 2:** Effect of boric acid (BA) treatments on the survival of Nile tilapia experimentally infected with S. parasitica.

	Tested BA concentrations in g/L									
	0		0.2		0.4		0.6		0.8	
	No. of Dead fish	Severity of infection	No. Dead fish	Severity of infection	No. Dead fish	Severity of infection	No. Dead fish	Severity of infection	No. Dead fish	Severity of infection
Day 1	1	non	0		0		0		0	
Day 2	0		0		0		0		0	
Day 3	5	moderate	0		0		0		0	
Day 4	1	severe	1	non	0		0		0	
Day 5	2	severe	2	mild	0		1	non	0	
Day 6	2	severe	2	mild	1	non	0		1	non
Day 7	1	severe	1	mild	0		0		0	
Day 8	3	severe	0		1	non	0		1	non
Day 9	2	severe	1	mild	0		2	non	0	
Day 10	3	severe	1	mild	1	non	0		0	

Severity of lesions associated with experimental infection is also presented as follows: non, for no lesions (absence of mycelial growth); mild, for scanty mycelial growths on fish body; moderate, for obvious mycelial growth affecting single area and severe for multiple obvious mycelial growth.

**Table 3:** The mortality (m, 0 = alive, 1 = dead) over the course of the experiment is summarized below for the different concentrations of boric acid.

m	Concentration (BA; g/L)					
	0.0	0.2	0.4	0.6	0.8	Total
0	10	16	27	27	28	108
1	20	14	3	3	2	42
Total	30	30	30	30	30	150

### ALT and AST enzymatic activity

**Figure 6:**
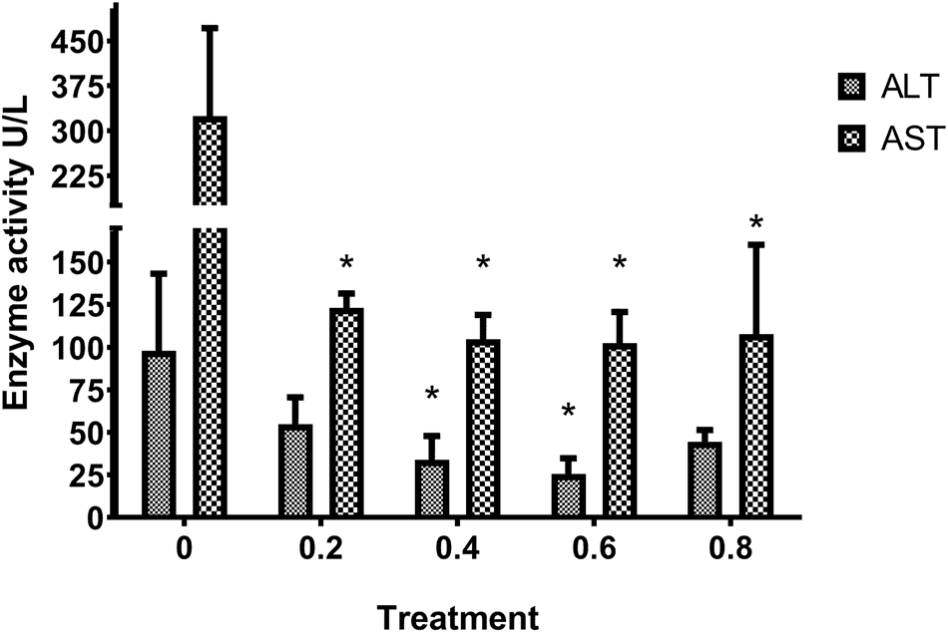
ALT and AST enzymatic activity in *Saprolegnia*-infected fish treated with different concentration of BA. Bars represent the average values obtained from three fish ± SD. Asterisks indicate statistical significance (*p < 0.05–0.01).

In addition to mortality, we also studied tissue damage induced by *Saprolegnia* infection under different BA treatments. Fish that were infected with *Saprolegnia* but not treated with BA exhibited the highest increase in both Alanine Aminotransferase (ALT) and Aspartate Aminotransferase (AST) enzymatic activity (Fig. 6). For BA treated fish, there was a significant (p < 0.05) reduction in AST levels for all tested concentrations compared to the non-treated control. A similar reduction was also observed for ALT but the difference was statistically significant (p < 0.05) only at treatment concentrations of 0.4 and 0.6 g/L (Fig. 6).

## Discussion

In the present study, we have shown that BA can be used as a preventive treatment against saprolegniosis in Nile tilapia, as previously demonstrated for fertilized eggs and yolk sac fry of Atlantic salmon^13^. No adverse effects were observed over 10 days of continuous exposure apart from mild genotoxic effect after 96 h continuous exposure to BA.

Boric acid is an essential nutrient for many living organisms including fish^14–16^ and can be used to control infections in humans^17^, animals and plants^18^. In the current study, we have evaluated the inhibitory effect of BA on *Saprolegnia* hyphal growth *in vitro* and found approximately 5% inhibition of *Saprolegnia* radial growth at the lowest concentration (0.1 g/L BA). A partial dose-dependent inhibitory effect that ranged from 22.5% to 80%, was observed in all tested concentrations below 0.6 g/L, while complete inhibition was achieved at a BA concentration ≥0.6 g/L. These findings are concordant with Ali *et al*.^13^, who reported similar effect of BA in controlling the growth and proliferation of two *Saprolegnia* spp. (*S. parasitica* and *S. diclina*) isolated from Atlantic salmon and their eggs. There are however some differences regarding *in vitro* sensitivity of the *S. parasitica* to BA. In the previous study^13^, we found 100% inhibitory effect at 0.7 g/L and above while in the present study, complete growth inhibition was obtained at 0.6 g/L and above. Although this difference could be viewed as a normal variation in experimental conditions at different laboratories, it can also be due to different isolates being tested. Indeed, Ali *et al*.^13^ showed differences in sensitivity to BA treatment between two *Saprolegnia* spp., however, such variations have not been evaluated in the present study because only one strain was tested. Additionally, *S. parasitica* isolates from Nile tilapia and Atlantic salmon showed different growth patterns at equal BA concentrations, with *Saprolegnia* isolate from tilapia showing higher growth rate. Besides origin of fish species, the variation in culture conditions included the use of SDA (for tilapia isolates) in contrast to glucose yeast (GY) agar used for the salmon isolates. A recent study has investigated the efficiency of some chemicals including BA against different *Saprolegnia* isolates^19^. The study has reported considerable reduction in the growth rate of BA treated mycelia and concluded that the minimal inhibitory concentration (MIC) of BA could be set in between two concentrations, 500 and 1000 ppm which is in accordance with our finding. The same study has confirmed the efficacy of other tested chemicals against *Saprolegnia*, however, none of them have been tested under *in vivo* conditions except for hydrogen peroxide which is known for its ability to control saprolegniosis on eggs of rainbow trout and chinook salmon^9,20,21^ though, much higher concentration is needed (5000 ppm).

Although several chemicals have been investigated for their ability to control saprolegniosis^22^, most of them were tested under *in vitro* conditions with limited *in vivo* trials. In catfish, formalin prevented *Saprolegnia* infection at concentrations of 12.5 and 25 mg/L; resulting in 86.7 and 96.6% survival respectively^23^. However, neither the presence of *Saprolegnia* infection-related lesions nor adverse effects were investigated in surviving fish. The same study reported 100% mortality in catfish treated with copper sulphate at a concentration of 0.5 mg/L. Bronopol is effectively protecting rainbow trout from *Saprolegnia* infection at concentrations of 10, 15 and 20 mg/L; resulting in 62% survival at the lowest tested dose (10 mg/ml) and 100% survival at 15 and 20 mg/L^5^, however, its adverse effects on surviving fish have not been investigated. Nile tilapia treated with potassium permanganate (100 mg/L) or hydrogen peroxide (420 mg/L) exhibited 67 and 63% survival rate respectively^24^. Nevertheless, presence of *Saprolegnia* lesions were not investigated and no focus was put on investigating the adverse effect although some liver enzymes levels were tested. The adverse effects of using the potassium permanganate were addressed in a separate study using much lower concentrations^25^. In the current study, the *in vivo* experiment included testing presence of *Saprolegnia* lesions after treatment and possible adverse effects associated with the treatment. The results obtained revealed that BA has protective effect against *Saprolegnia* infection in all treated groups except at 0.2 g/L, where mild signs of saprolegniosis were observed. However, the survival rate of BA treated fish (0.2 g/L) was relatively high (73.3%) compared to survival in non-treated control (33.3%) which exhibited moderate to severe signs of infection. In addition, no signs of toxicity or abnormality were observed over a 10 days exposure. The high survival rates and the absence of gross abnormalities of treated fish is an indication of safety. It is difficult to make a direct comparison between our study and the other studies mentioned above because of the different type of chemicals and fish species used. The study conducted by Ali *et al*.^13^ is the most relevant since it has reported similar findings in yolk sac fry hatched from BA-treated eggs. This is in contrast to the spinal, head, fin and tail abnormalities reported in trout fry hatched from eggs treated with malachite green^26,27^

In addition to the *in vitro* and *in vivo* testing, the safety of BA as a treatment for Nile tilapia was evaluated using different concentrations. Malachite green which has well demonstrated toxic effects^6,28^ was used as a positive control treatment for assessment of toxicity. At 3 g/L BA, cumulative mortality was 50% following 60 h exposure. Based on the results obtained following 96 h continuous exposure to several BA concentrations, the LC_50_ was estimated to be 1.56 g/L for Nile tilapia with an average body weight of 22 g. In addition, the genotoxic effects of BA were investigated using the comet assay. This test has been used extensively to monitor the level of toxicants in water using many fish species including tilapia^29^. Based on the *in vitro* results, samples were collected from tilapia treated at BA concentrations of 0.6 g/L (yielding complete inhibition of growth) and below, in addition to controls. Compared to the non-treated groups, only 2% increase in the percent of damaged DNA was reported in BA treated groups compared to 6% increase in MG treated ones. No histomorphological changes were observed at tested BA concentrations between 0.2 g/L and 0.6 g/L. Our findings therefore suggest that therapeutic levels of BA could cause mild genotoxicity but with no negative impact on the viability of treated tilapia fish. It must be noted, however, that the toxic effect of BA was monitored under 96 h continuous exposure while treatments are usually applied for much shorter time. The toxic effect is therefore expected to be lower under standard treatment conditions. Our data suggests that the optimal therapeutic concentration to be used may be 0.6 g/L since no infection was reported from this concentration and above.

Additionally, assessment of any negative impact of BA treatment also included measurement of AST and ALT serum levels. Both enzymes are used as indicators of tissue damage^30^ and their levels were also shown to be increased upon exposure to toxic materials in many fish as shown in some fish species following exposure to cadmium & copper sulfate^31,32^. High levels of these enzymes coincided with severe and moderate infection in non-treated control fish while relatively low levels were detected in BA-treated groups, including the 0.2 g/L group with mild infection. This indicates that BA decreases the systemic impact of *Saprolegnia* infection, and presumably reduces stress in infected fish. It must be noted that it was not possible for us to conclude whether AST and ALT were within normal limits or not since uninfected untreated controls were not included. Interestingly, higher increases of AST was detected compared to ALT. This is in contrast to the general notion that ALT is more responsive to tissue damage^30^ and should be further studied in relation to pathogenicity. With regard to tissue damage, our findings indicate that the damage induced by *Saprolegnia* infection exceeds the adverse effects by prolonged exposure to therapeutic levels of BA. All obtained data indicate that BA is efficient and safe for use for preventing *Saprolegnia* infection in Nile tilapia. The main limitation when it comes to practical applications is that we only tested prolonged, continuous exposure in the present study which is somewhat impractical. Follow-up studies should therefore be conducted to determine the optimal (shortest) timing for BA exposure to prevent *Saprolognia* infection in the field, and if intermittent treatment can be used (for example 1 time per day). Furthermore, to what extent can BA be used to treat already infected tilapia should also be explored in more detail.

The *Saprolegnia* isolate used in the current study was identified as *S. parasitica*, also previously shown by Zahran *et al*.^4^ to cause infection in Nile tilapia. Zahran *et al*.^4^ suggest the presence of different *S. parasitica* strains as a cause of saprolegniosis in Nile tilapia in Egypt. Future studies should explore strain differences for *S. parasitica* isolates from Egypt, including possible differences in sensitivity to BA.

## Materials and Methods

### *Saprolegnia* strain

*Saprolegnia* sp. used in this study was isolated from a natural outbreak of saprolegniosis in Nile tilapia with more than 70% reported mortalities. Isolation was performed as described by^12^, by extracting the *Saprolegnia* from the skin of infected fish followed by inoculation on SDA containing antibiotics (200 µg mL^−1^ chloramphenicol) for 24 hours. After 24 hours incubation, a small part of agar with emerging hyphal tip was transferred to fresh media in order to obtain a clean culture. Subsequently, a piece of the clean culture was incubated in autoclaved pond water for production of zoospores and single spore culture was performed on SDA. Purified isolate was identified by PCR and sequencing using universal fungal primer ITS1-ITS4 as described previously^33^. Following sequencing, sequence alignment and phylogenetic analysis was performed using an online software^34^. The isolates used to perform the analysis and their accession numbers are provided in supplementary table S1.

### *In vitro* activity of boric acid on *Saprolegnia* hyphal growth

The *in vitro* activity of boric acid against the growth of *Saprolegnia* hyphae was evaluated according to the method described by Beakes & Gay^35^. Briefly BA (Sigma-Aldrich) was dissolved in sterilized distilled water (SDW) and then incorporated at different concentrations into molten SDA held at 45 °C. The concentrations were 0.1, 0.2, 0.3, 0.4, 0.5, 0.6, 0.7, 0.8, 0.9 and 1.0 g/L. Sterile distilled water without BA was used as negative control treatment (0.0 g/L). Agar plugs (2 mm) with actively growing *Saprolegnia* mycelia were then inoculated in the center of SDA plates and the average radial growth of *Saprolegnia* hyphae was measured following 72 h incubation. All the concentrations were tested in triplicates.

### Ethics statement

The experiment was carried out in accordance with “Guidelines for the Use of Fishes in Research” published by American Fisheries Society (2014). Moreover, the research work was done by the first author who completed Laboratory Animal Science Course for Research workers which satisfies the requirements of the Norwegian Ministry of Agriculture and Food’s definition of competence at “FELASA C” level. Additionally, the research work was strictly supervised by advisory committee with the approval of Worldfish Egypt country director.

### Assessment of BA safety in tilapia

In order to determine the suitability and safety of BA for use in Nile tilapia, we studied the toxicological effect after exposure to different BA concentrations for 96 h continuously. For this purpose, 180 farmed tilapia with average body weight 22 ± 3 g were used. Fish were divided into 9 main groups consisting of 20 fish and each of the main groups was further sub-divided into two groups placed in separate tanks (exposure in duplicate). Seven BA concentrations were tested in addition to controls. Tested BA concentrations were 0.2, 0.4, 0.6, 0.8, 1.0, 2.0, and 3.0 g/L. Aquarium water was used as a negative control treatment for toxicity while MG was used at a concentration of 0.5 mg/L^36^ as a positive control for toxicity. Mortality was recorded at 12, 24, 36, 48, 60, 72, and 96 h post exposure. At the end point (96 h), the cumulative mortality was recorded in all groups and the LC_50_ was determined using online software (http://www.ic50.tk). In addition, blood samples were collected from the groups exposed to 0, 0.2, 0.4, and 0.6 g/L as well as from moribund fish exposed to malachite green. The samples were collected by severing the caudal vein using syringes and transferring immediately to collection tubes containing anticoagulants for nucleic acid damage examination using a comet assay test.

### Assessing DNA damage using comet assay

Comet assay was performed according to the method described by Singh *et al*.^37^, using a three step protocol followed by immersion in lysis buffer, electrophoresis and finally staining with ethidium bromide. Two slides were prepared for each BA concentration and controls. DNA damage was assessed by calculating the percent damaged DNA in a total of 100 counted nuclei. The analysis was done blindly without prior knowledge of the sample nature.

### Histopathology

Samples from gills, liver and kidney of BA treated groups (0.6 g/L and below) and controls were collected for histopathological examination. All samples were fixed in 10% buffered formalin. Histopathology sections were stained with haematoxylin and eosin (H&E) after processing using standard laboratory procedures.

### Efficacy of BA in preventing *Saprolegnia* infection in Nile tilapia

About 150 Nile tilapia fish with average body weight 50 ± 5 g were used for the *in vivo* testing. Fish were divided into five main groups, four for BA and one for non-treated negative control. Each main group consisting of 30 fish was further subdivided into three replicates of 10 fish each, placed in separate tanks. Based on the toxicity data obtained, four BA concentrations corresponding to doses equal or below half of the estimated LC_50_ were tested for their ability to prevent *Saprolegnia* infection in Nile tilapia. Tested BA concentrations were 0.2, 0.4, 0.6, and 0.8 g/L. For zoospore production, method described by Stueland *et al*.^12^ was followed. Briefly, bundles of growing Saprolegnia hyphae were washed twice in autoclaved pond water (APW), transferred to glass bottle containing APW and incubated at 21 °C for 24 h to allow extensive zoospore production. The zoospore suspensions were filtered through sterilized tea filter (0.5 mm pores), a procedure that is expected to result in zoospore encystment. Obtained cysts were counted using a haemocytometer (Bürkertürk chamber). Fish were subjected to “ami-momi” treatment as described previously by Hatai & Hoshiai^38^ and Stueland *et al*.^12^ before being exposed to *Saprolegnia* spores at a concentration of 1.0 × 10^4^ spores L^−1^. Except for the control, boric acid was added at the time of infection, to the tanks to reach the corresponding concentrations. All treated groups were exposed to BA continuously for ten days. Water was not exchanged over this period and the fish were not fed. Over this period, fish were observed for signs of *saprolegniosis*. Number of dead fish, temperature and pH were recorded daily. At the end of the 10-day period, blood samples were collected by severing the caudal vein using syringes as described previously. Fish were euthanized using overdose of Tricaine Methanesulfonate (MS-222, Sigma-Aldrich) in water. Serum was separated from the blood and the activities of ALT and AST enzymes were measured using DRI-CHEM NX500 VET analyzer and slides.

### Statistical analysis

Kurskal-Wallis test was performed to determine differences in DNA damage induced using GraphPad prism 8. One-way ANOVA followed by Dunnett’s multiple comparisons test was also performed using GraphPad Prism version 8.0.0 for Windows, GraphPad Software, San Diego, California USA, for differences in radial growth of Saprolegnia at different boric acid concentrations. Similarly, differences in ALT and AST was performed using the same method. A log-rank test for equality of survivor function was carried out for the challenge experiment using different BA concentrations, followed by a Cox hazard ratio estimation. Differences between treated and controls were evaluated at a p-value < 0.05.

## Supplementary information

Supplementary doc
