# Supplementary material for: Efficacy and safety of boric acid as a preventive treatment against Saprolegnia infection in Nile tilapia (Oreochromis niloticus)
Source: Sci Rep. 2019 Nov 29;9:18013. doi: 10.1038/s41598-019-54534-y (PMC6884594; doi:10.1038/s41598-019-54534-y)
Supplement: Supplementary file 1 — Supplementary doc [file 41598_2019_54534_MOESM1_ESM.docx]

**Efficacy and safety of boric acid as a preventive treatment against *Saprolegnia* infection in Nile tilapia (*Oreochromis niloticus*)**

Shimaa E. Ali^1, 2*^, Amr A.A. Gamil^3^, Ida Skaar^4^, Øystein Evensen^3^, Harrison Charo-Karisa^1^

^1^Worldfish, Egypt

^2^Department of Hydrobiology, National Research Centre, Dokki, Giza, Egypt

^3^ Faculty of Veterinary Medicine, Norwegian University of Life Sciences, Oslo, Norway,

^4^Norwegian Veterinary Institute, Oslo, Norway

^*^Correspondence and requests for materials should be addressed to Shimaa E. Ali (email: [Shimaa.ali@cgiar.org](mailto:Shimaa.ali@cgiar.org))

**Table S1.** Accession numbers for the ITS rRNA gene sequences used to perform the phylogenetic analysis

| **Strain** | **accession no.** |
| --- | --- |
| Saprolegnia parasitica strain SAP1381 | KF717876.1 |
| Saprolegnia ferax strain SAP1234 | KF717954.1 |
| Saprolegnia delica strain SAP1524 | KF718043.1 |
| Saprolegnia australis strain SAP1105 | KF718010.1 |
| Saprolegnia diclina strain SAP1281 | KF717815.1 |
| Saprolegnia litoralis strain SAP1486 | KF718048.1 |
| Saprolegnia monilifera strain SAP1450 | KF718131.1 |
| Saprolegnia torulosa strain SAP0856 | KF718125.1 |
| Saprolegnia terrestris strain SAP1285 | KF718134.1 |
| Saprolegnia subterranea strain SAP1293 | KF718124.1 |
| Achlya sp. SCAAD | GU014261.1 |
| Achlya sp. O3EG1 | GU014271.1 |
| Saprolegnia eccentrica strain SAP1288 | KF718140.1 |
| Saprolegnia furcata strain SAP1294 | KF718143.1 |
| Saprolegnia anisospora strain SAP1295 | KF718201.1 |
| Saprolegnia anisospora strain SAP0270 | KF718193.1 |
| Saprolegnia turfosa strain SAP1279 | KF718190.1 |
| Protoachlya paradoxa strain SAP1873 | KF718202.1 |
| Saprolegnia monoica voucher CBS59967 | HQ643998.1 |
| Saprolegnia asterophora strain SAP1296 | KF718178.1 |
| Leptolegnia sp. SAP0772 | KF718185.1 |
| Achlya caroliniana strain SAP1240 | KF718203.1 |
| Saprolegnia racemosa voucher RJBCC0018 | KR872862 |
| Saprolegnia racemosa voucher RJBCC0019 | KR872863 |


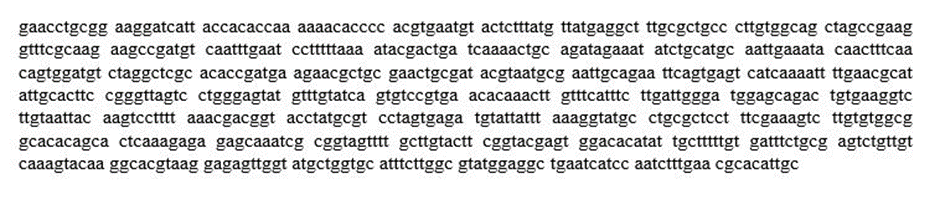


**Figure S1.** Sequence of ITS rRNA gene obtained from Saprolegnia isolated from Nile tilapia.


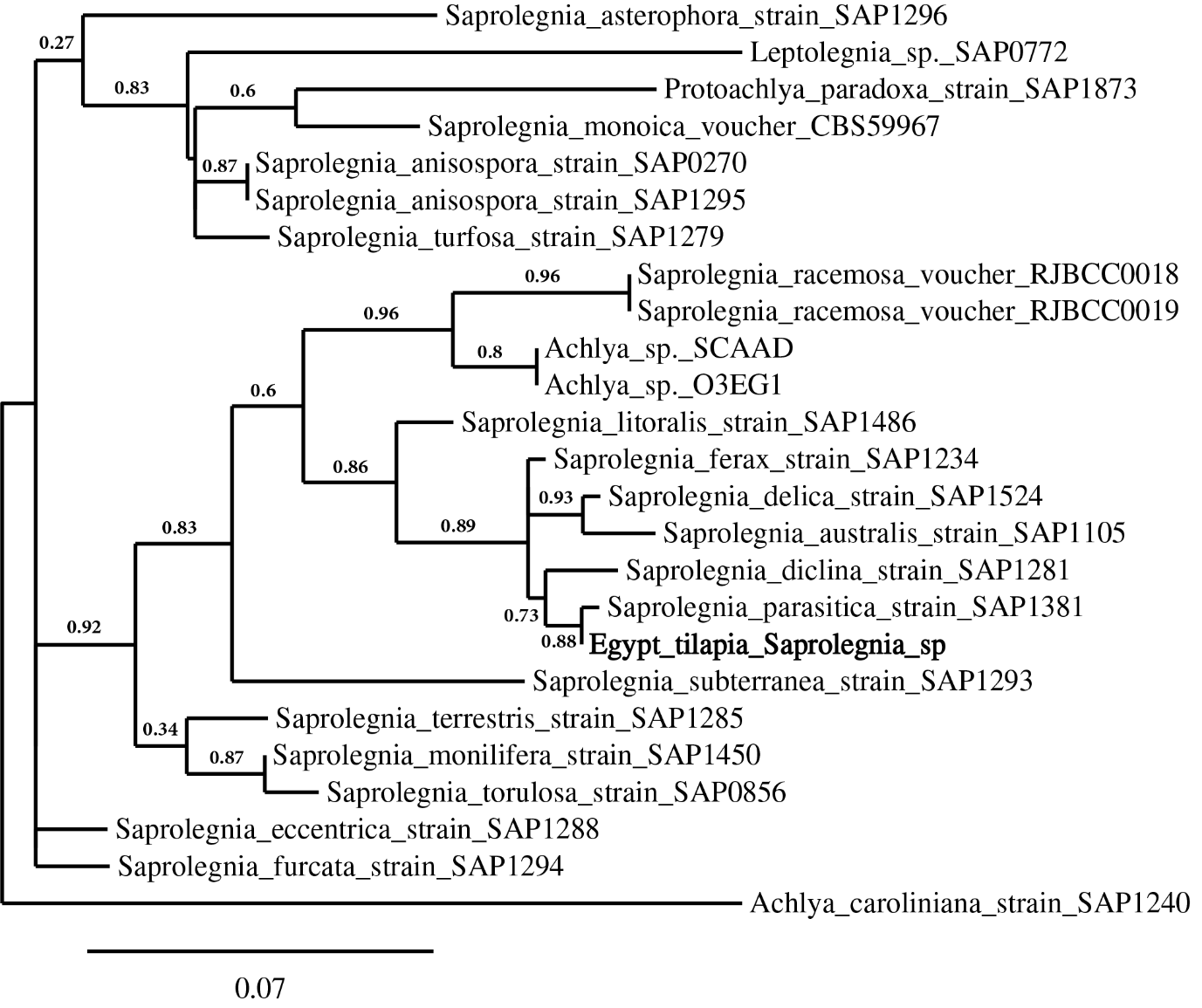


**Figure S2.** Phylogenetic tree showing the relation of the isolated Saprolegnia from Nile tilapia (bold) to the other isolates.


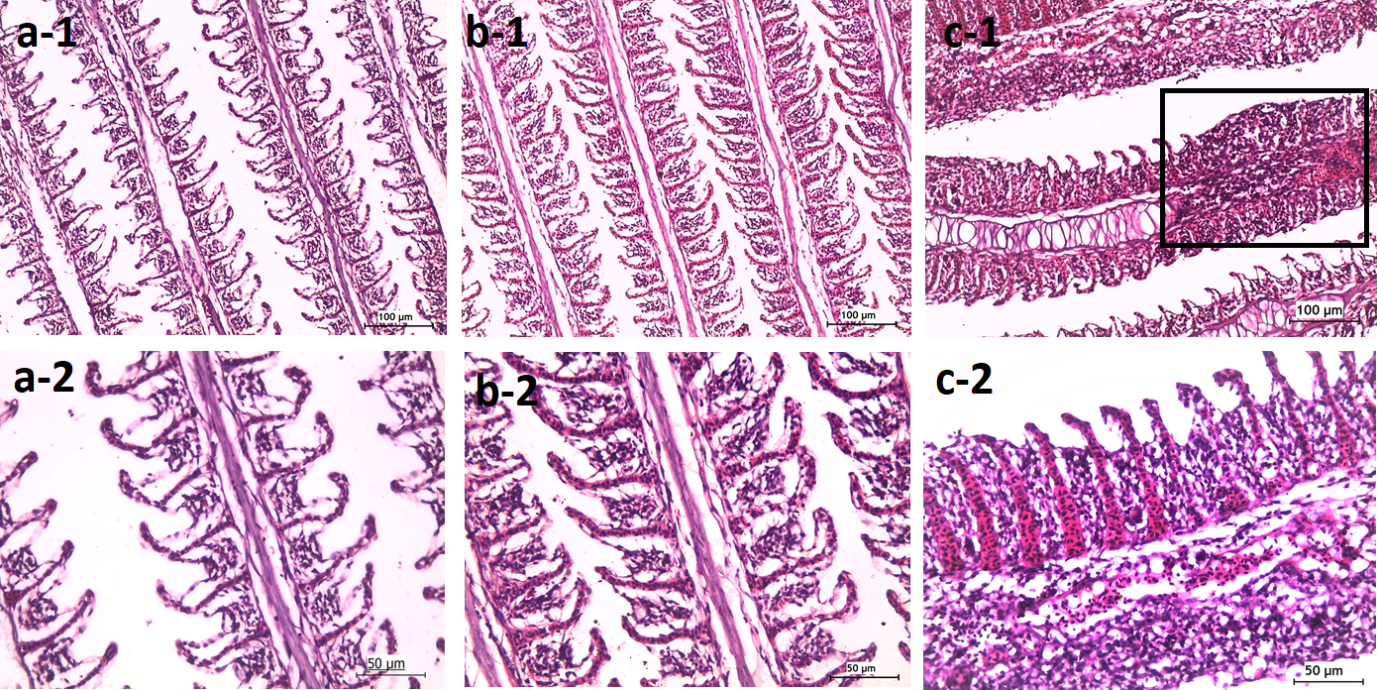


**Figure S3.** Micrographs of gills of Nile tilapia treated with boric acid (b) (0.6 g/L) and malachite green (c). Non-treated control in a-1 and a-2. No obvious difference between treated and controls apart from an area with cellular hyperplasia in c-1/c2. These are more chronic changes caused by other factors than exposure to malachite green. Moderate post-mortem changes.

**
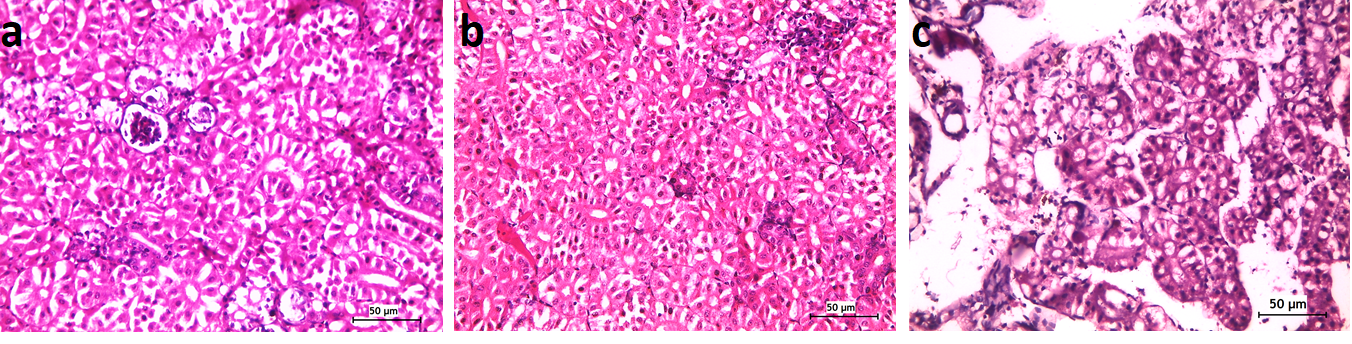
**

**Figure S4.** Micrographs of kidney of Nile tilapia treated boric acid (b) (0.6 g/L), malachite green (c), compared to non-treated control (a). There are no obvious changes that can be associated intra vitam pathological processes.

**
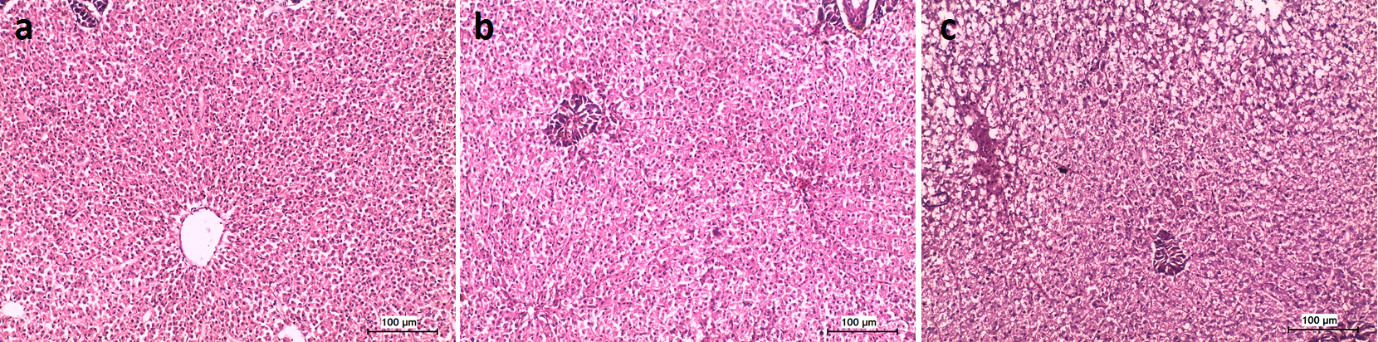
**

**Figure S5.** Micrographs of liver of Nile tilapia treated boric acid (b) (0.6 g/L), malachite green (c), compared to non-treated control (a). There are no obvious changes that can be associated intra vitam pathological processes.
